# Supplementary material for: MCL1 Inhibition Overcomes the Aggressiveness Features of Triple-Negative Breast Cancer MDA-MB-231 Cells
Source: Int J Mol Sci. 2023 Jul 6;24(13):11149. doi: 10.3390/ijms241311149 (PMC10342057; doi:10.3390/ijms241311149)
Supplement: Supplementary file 1 [file ijms-24-11149-s001.zip › Supplementary Materials-Captions.pdf]

Figure S1. Serum deprivation enhances 10 μM A-1210477 effect on MDA-MB-231 cell viability. (**A**) Phase-contrast microscopy images of MDA-MB-231 cells showing the effects of A-1210477 treatment on cell morphology (magnification 200X) in presence or absence of Fetal Bovine Serum (FBS) in culture medium. Cells were treated with 10 μM A-1210477 for different time (2-72 h) of incubation. Anoikis was induced after 2 h of treatment in FBS deprivation respect with the control cells. Notably, serum deprivation did not induce cell death in untreated cells, showing only proliferation slowdown. (**B**) MDA-MB-231 cell viability was performed by MTT assay and expressed as the percentage with respect to control cells. Cell viability was drastically reduced after 2 h of 10 μM A-1210477 treatment in FBS deprivation respect with FBS presence. All the experiments were performed in triplicate. Data are expressed as mean ± SD. ND (Not Detectable); ***$p < 0.001$.

Figure S2. Apoptotic effect induced by A-1210477 in MDA-MB-231 cells. Flow cytometry analysis by Annexin V and PI staining was performed to detect apoptotic ratio in MDA-MB-231 cells following 10 μM A-1210477 treatment (72 h). The staining fluorescent protocol was performed in accordance whit the manufacturer instructions (Annexin V-FITC Kit, Miltenyi Biotec, Germany). Briefly, cells were trypsinzed, centrifuged, resuspended in Binding Buffer (BB) and counted to define cell number. Then, cells were washed twice and resuspended in 100 μL/10^6 cells of BB and labelled with 10 μL of FITC-Annexin V for 15 minutes in the dark. At the end of incubation, cells were washed twice and resuspended in 500 mL/10^6 cells in 1x BB with 5 mL of Propidium Iodide (PI) immediately prior to analysis. Flow cytometry analyses were performed by a FACSCanto TMII (BD Biosciences) and FlowJo v10 software. Double labelling with Annexin V and PI allows a distinction of early apoptotic (Annexin V^+/PI^-), late apoptotic (Annexin V^+/PI^+) and viable (Annexin V^-/PI^-) cells.

Table S1. List of antibodies used.

Table S2. List of qRT-PCR primers used.
