# Supplementary figures and images for: MCL1 Inhibition Overcomes the Aggressiveness Features of Triple-Negative Breast Cancer MDA-MB-231 Cells
Source: Int J Mol Sci. 2023 Jul 6;24(13):11149. doi: 10.3390/ijms241311149 (PMC10342057; doi:10.3390/ijms241311149)

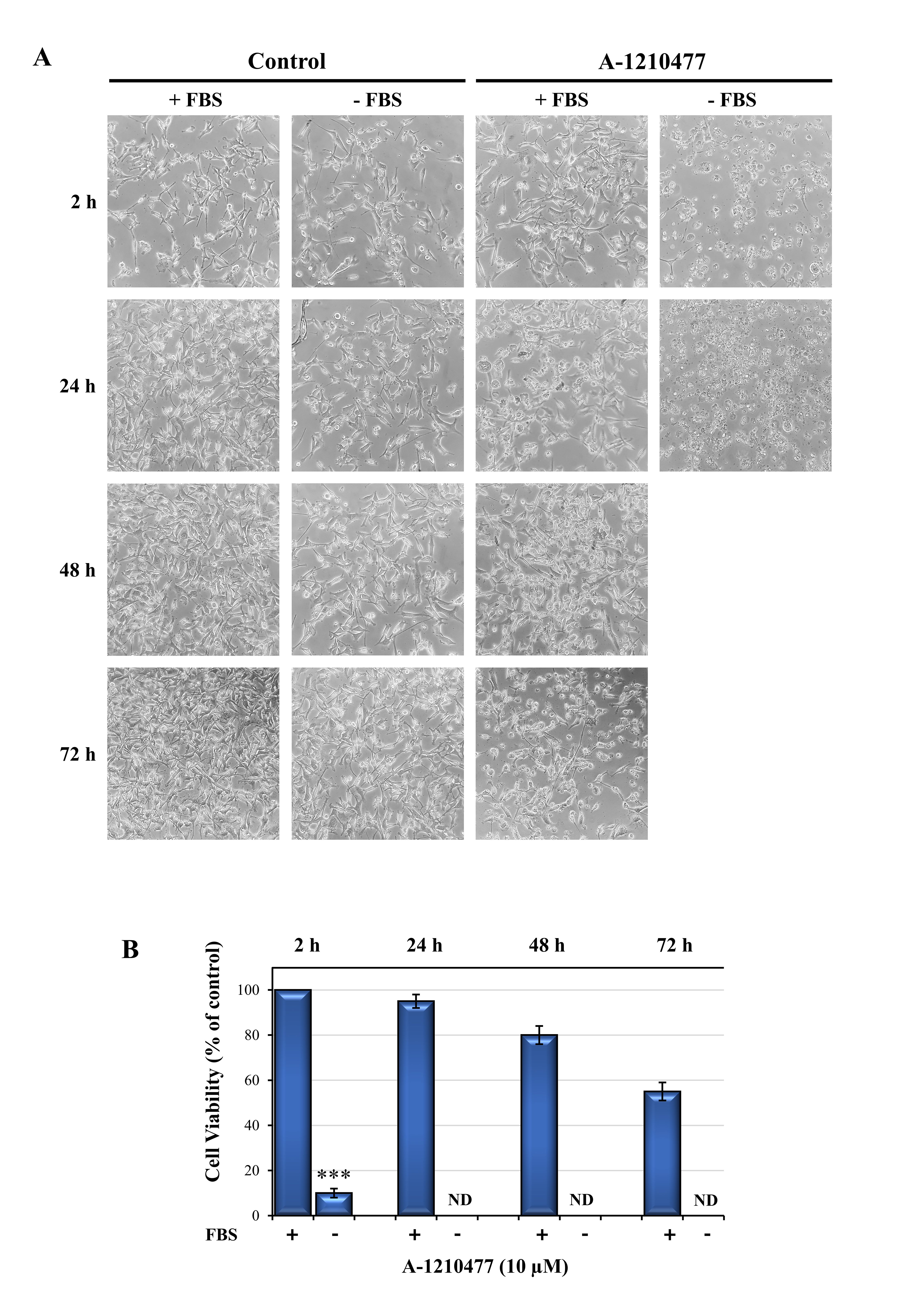

Supplement: Supplementary file 1 [file ijms-24-11149-s001.zip › Figure S1.tif]

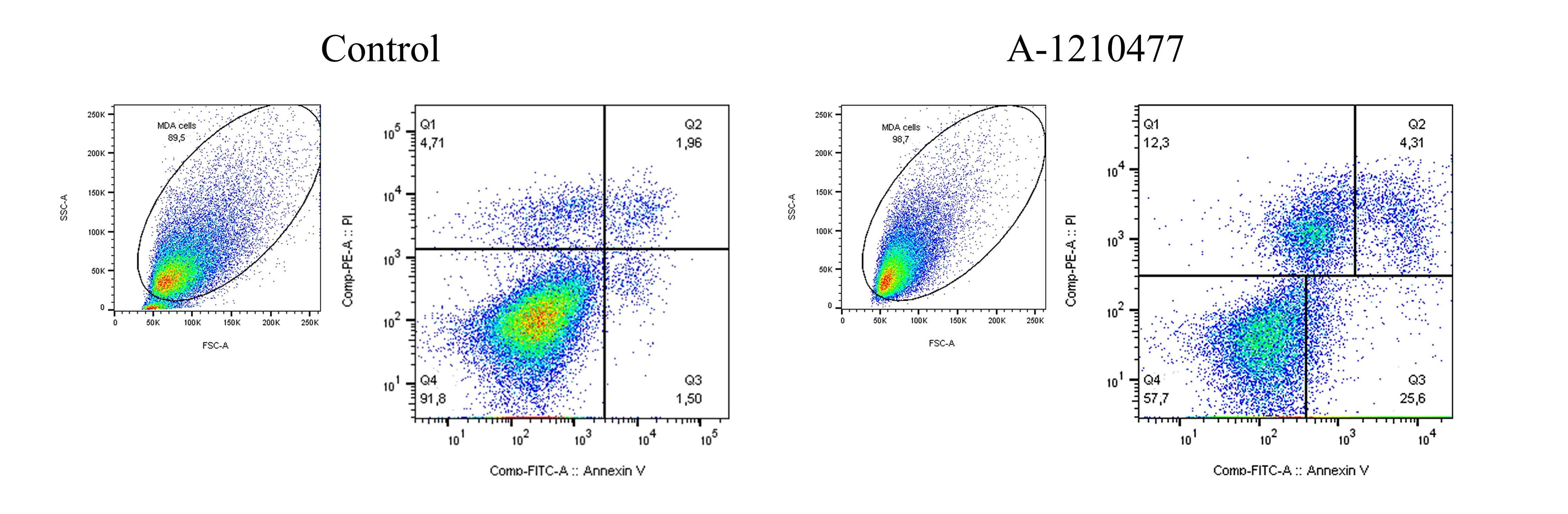

Supplement: Supplementary file 1 [file ijms-24-11149-s001.zip › Figure S2.tif]

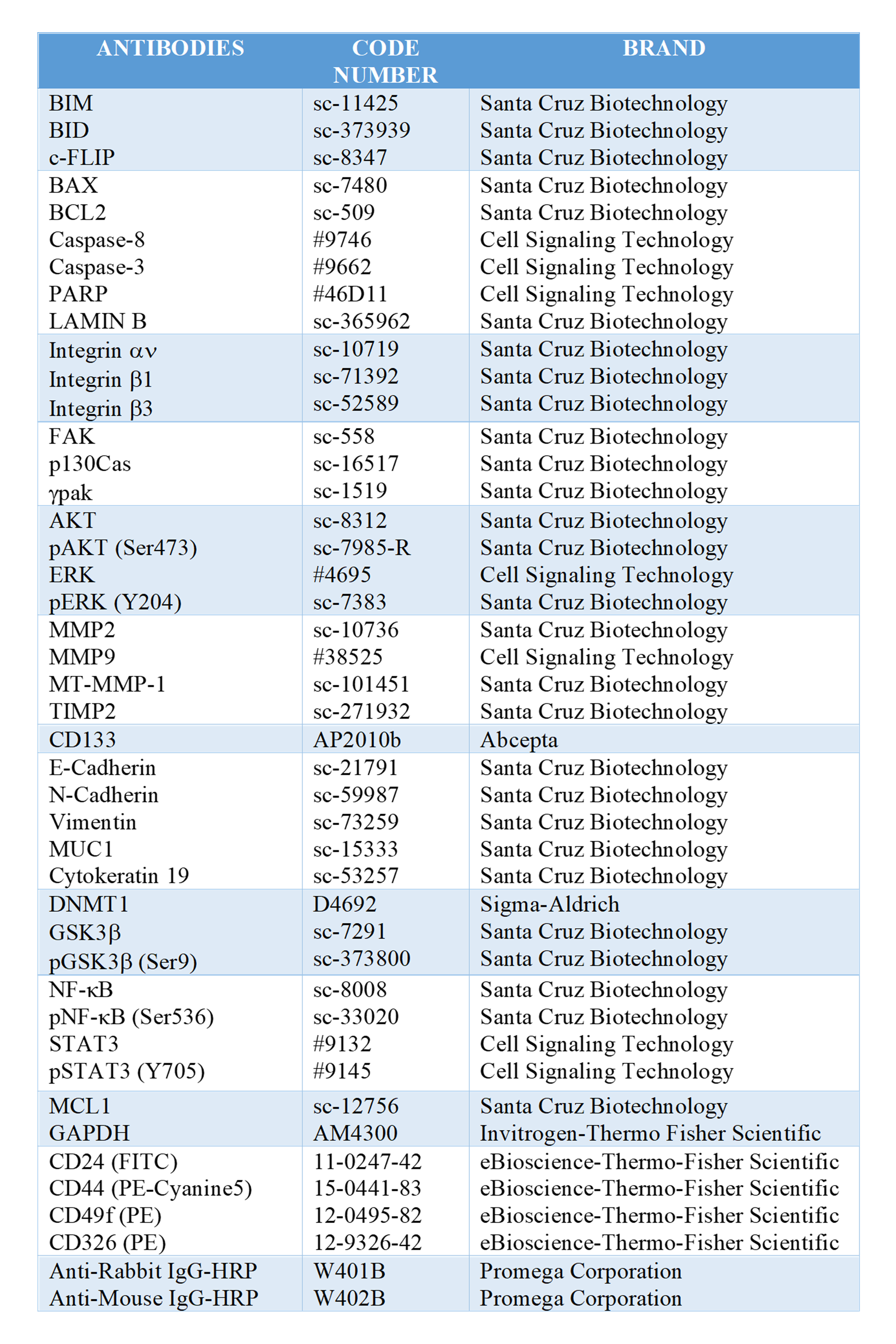

Supplement: Supplementary file 1 [file ijms-24-11149-s001.zip › Table S1.tif]

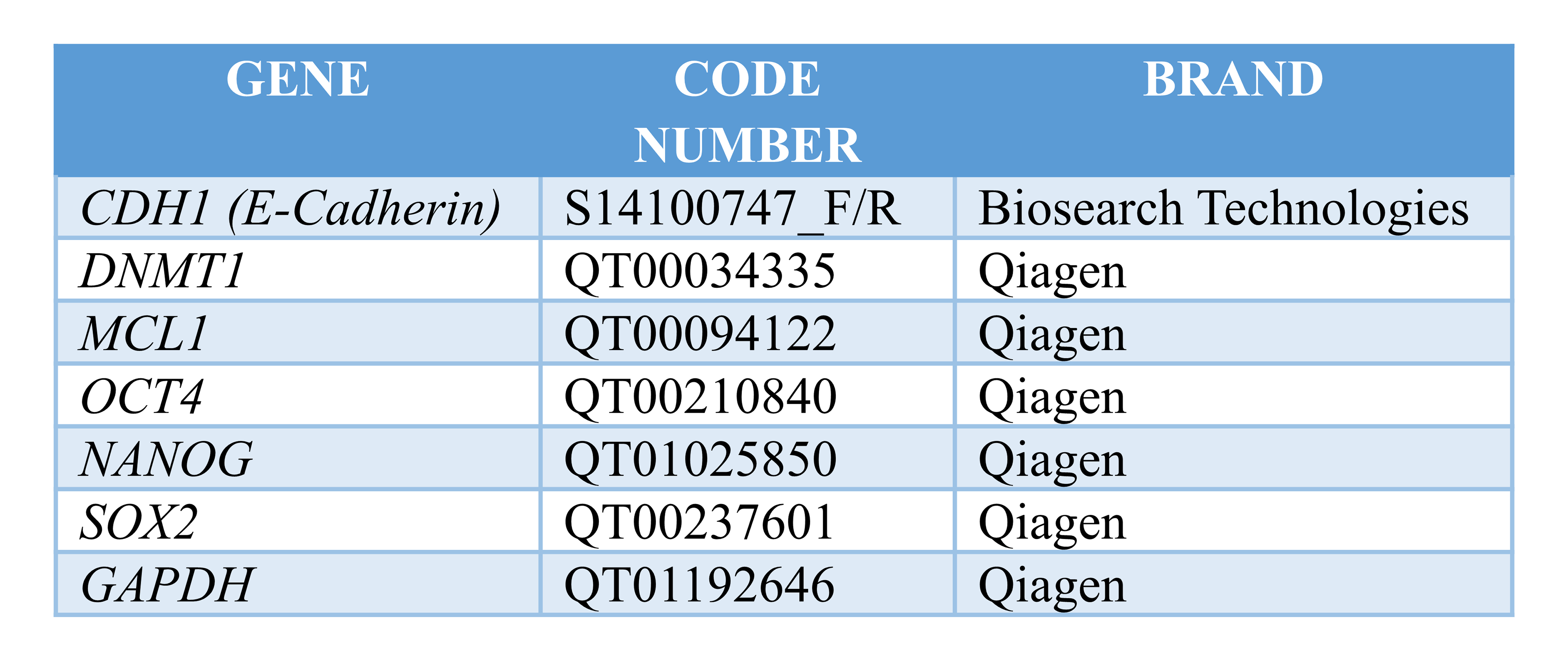

Supplement: Supplementary file 1 [file ijms-24-11149-s001.zip › Table S2.tif]
